# Degradation of Poly(ε-caprolactone) Resorbable Multifilament Yarn under Physiological Conditions

**DOI:** 10.3390/polym15183819

**Published:** 2023-09-19

**Authors:** Monica V. Deshpande, Arjunsing Girase, Martin W. King

**Affiliations:** 1 Wilson College of Textiles, North Carolina State University, Raleigh, NC 27606, USA; aggirase@ncsu.edu; 2 College of Textiles, Donghua University, Shanghai 201620, China

**Keywords:** Poly(ε-caprolactone), biodegradable, physiological degradation, biomedical textiles, tissue engineering scaffold

## Abstract

Poly(ε-caprolactone) (PCL) is a hydrophobic, resorbable aliphatic polymer recognized for its low tenacity and extensive elongation at break, making it a popular choice for fabricating biodegradable tissue engineering scaffolds. PCL’s slow degradation rate typically results in a complete resorption period of 2 to 3 years. While numerous studies have examined the degradation of PCL in various forms such as films and webs, no study to date has investigated its physiological degradation in multifilament yarn form. In this study, we subjected PCL multifilament yarn samples to physiological conditions in phosphate-buffered saline (PBS) maintained at a consistent temperature of 37 ± 2 °C and agitated at 45 rpm for a period of 32 weeks. We retrieved samples at five different intervals to analyze the degradation profile of the multifilament yarn. This allowed us to estimate the complete resorption time and rate under these in vitro conditions. Over the 32-week period, the multifilament yarn’s mass decreased by 4.8%, its elongation at break declined by 42%, the tenacity dropped by 40%, and the peak load at break fell by 46.5%. Based on these findings, we predict that a scaffold structure incorporating PCL multifilament yarn would undergo complete resorption in approximately 14 months under physiological conditions, such as in PBS solution at a pH of approximately 7 and a temperature of 37 °C.

## 1. Introduction

Poly(ε-caprolactone) (PCL) is a semicrystalline, highly elastomeric, biodegradable, and bioresorbable polymer characterized by a glass transition at −60 °C and a melting point at +60 °C [1]. The polymer is synthesized from the ring-opening polymerization of ε-caprolactone. PCL is biocompatible, approved by the FDA as the polymer for fabricating certain implantable devices, and distinguished by its hydrophobicity, low tensile strength (approximately 23 MPa), and high elongation at break (700%). It is a slow-degrading polymer, typically requiring 2 to 3 years for complete degradation. Hence, it is often used as a copolymer with other degradable polymers such as polylactic acid (PLA) and polyglycolic acid (PGA) so as to reduce the level of crystallinity and increase the rate of degradation. It is also often blended with biological materials, like hydroxyapatite and collagen. Its ease of manufacturing allows PCL to be extensively utilized in fabricating biodegradable scaffolds for craniofacial tissue regeneration using electrospinning and 3D printing techniques [2,3].

Degradation studies are critical for evaluating the degradation profile of polymeric structures and estimating the time required for complete degradation or resorption following implantation in the body as a tissue engineering scaffold. Polymeric biomaterials can degrade via several mechanisms, including hydrolytic or oxidative degradation [4]. The ‘functional time’, or the period the structure maintains its desirable function, and the ‘disappearance time’, which is the period for the structure to completely resorb and lose its mass, can be used to determine the degradability of biocompatible polymers [4]. Various degradation studies have been undertaken, both in vitro and in vivo, over different periods of time so as to determine the suitability of such polymers for specific applications in tissue engineering or drug delivery [4].

For instance, Yoon et al. carried out a 180-day degradation study, both in vitro and in vivo, to assess the biocompatibility of a biodegradable cylindrical PLLA mesh [5]. They subjected PLLA biomaterial to degradation in PBS solution in the presence of lysozyme to observe changes in mass and tensile strength in vitro. For in vivo degradation, they implanted PLLA mesh in rats for 180 days and retrieved them at various time points to analyze the in vivo degradation profile through changes in mass and tensile strength [5].

In a different study, Yeo et al. evaluated the in vitro degradation of an 80/20 poly(ε-caprolactone)/tricalcium phosphate (PCL–TCP) scaffold in Dulbecco’s modified Eagle media (DMEM), measuring changes in porosity, pH, physical state, and mechanical properties [6].

As Labet et al. noted, the hydrolytic degradation profile of PCL based on its molecular weight distribution initially involves the degradation of the amorphous phase, which causes the level of crystallinity to increase while maintaining the molecular weight [7]. This is followed by the cleavage of the ester bonds, resulting in the loss of mass. At higher temperatures, the polymer degrades through a different mechanism of end-chain scission [7].

Previous research has examined PCL degradation in various forms, including films, monofilaments, copolymer structures, and polymer meshes [6,7,8,9,10,11]. However, the degradation profile of PCL in its multifilament form remains unexplored. Multifilament yarns offer distinct mechanical and surgical advantages, including high flexibility, ease of knotting, and improved knot security, over their monofilament counterparts. These properties make them more suitable for certain biomedical applications, such as the production of bioresorbable sutures and woven or knitted scaffolds for tissue engineering. It is, therefore, critical to understand the degradation behavior of PCL in multifilament yarn form to better predict its performance and lifespan in these applications.

Recognizing this knowledge gap, our study was designed to monitor the degradation profile of PCL multifilament yarns under physiological conditions for a period of 8 months. Through tracking the loss in mass and the changes in ultimate strength and elongation at break, our research aims to provide valuable insights into how PCL multifilament yarns can be used in tissue engineering and other biomedical applications. These data can serve as a baseline for predicting the lifespan of future biomaterials incorporating PCL multifilaments, ultimately improving their design and functionality in clinical applications.

## 2. Materials and Methods

### 2.1. Procurement of Yarn and Sample Preparation

**Figure 1:**
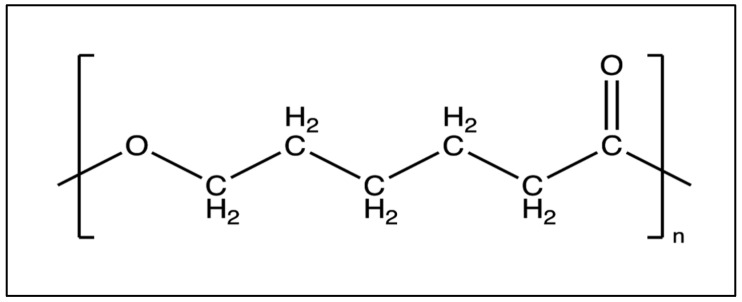
Chemical structure of PCL polymer.

The primary material used in this study was a bobbin of bioresorbable, 100% poly(ε-caprolactone) (PCL) multifilament yarn. This was sourced from Guangdong Zhuhai Adhesive Products Co., Ltd. (Zhuhai, Guangdong, China). This PCL yarn was spun from a semicrystalline polymer with a high amorphous content. This contributes to its high elongation at break, which is a critical property for the purpose of this research. The physical properties of the PCL multifilament yarn, outlined in Table 1, underscore its relevance to the study’s objectives. The chemical structure of PCL is shown in Figure 1.

For experimental consistency, the PCL multifilament yarn was processed into individual test specimens. Each yarn specimen was cut precisely to a 5-inch length. This uniform length ensured that the surface area exposed to the degrading solution would be constant across all samples, thereby allowing for more accurate comparisons.

To optimize the yarn’s contact with the surrounding degradation solution during the study, each 5-inch specimen was carefully wound onto a styrene frame. This ensured that the entire surface area of the yarn was exposed to the solution, which enhanced the accuracy and precision of the degradation study. The specimens were weighed prior to being wound onto the frames. Their initial mass was recorded and used for comparison between samples and over time during the degradation study.

### 2.2. PCL Yarn Degradation Study

The styrene frames carrying the prepared PCL yarn samples were submerged individually in glass beakers. Each beaker was filled with 20 mL of Dulbecco’s phosphate-buffered saline (dPBS) solution, sourced from Gibco Life Technologies (Gaithersburg, Maryland). The beakers were then sealed with parafilm to prevent contamination and evaporation during the experiment.

For the degradation study, the sealed beakers were placed in a Boekel Grant ORS 200 shaker bath (Boekel Scientific, Feasterville, Pennsylvania). The bath was set to maintain a consistent shaking speed of 45 rpm and a temperature of 37 ± 2 °C. This setup ensured an even distribution of forces acting on the surface of the multifilament yarn samples.

To maintain the pH of the dPBS solution at a consistent level of 7.0 ± 0.5, the solution in each beaker was replaced with fresh dPBS every two weeks. This step was crucial in mimicking physiological conditions for the degradation study.

Five specimens were collected at each of four different time points, namely 8, 16, 24, and 32 weeks, and the degradation profile of the PCL yarn samples was determined. The mass, tensile strength, and elongation at the break of each specimen were measured at each retrieval time.

Upon removal from the dPBS solution, the specimens underwent a cleaning process to remove residual solution. They were thoroughly rinsed with deionized water and then air-dried under a hood for 30 min. This ensured that the measured properties were inherent to the yarn samples and not influenced by any remaining surface contamination.

### 2.3. Evaluation of Tensile Strength and Elongation at Break

Prior to mechanical testing, each yarn specimen was weighed in milligrams. This step was vital to ascertain the linear density or denier of the yarn, which is a standard unit of measurement in grams per 9000 m.

The mechanical properties, including the tensile strength or tenacity and percent strain at break, were evaluated using an MTS Q-test mechanical tester (MTS Systems Corp., Eden Prairie, MN, USA) with a 5-pound load cell, a 1-inch gauge length, and a crosshead speed of 300 cm/min. The peak load at break, expressed in gram-force (gf), and the percent elongation at break were recorded for each yarn specimen. The tenacity of the yarn samples was then calculated in terms of gram-force per denier. This was achieved through dividing the peak load at break (in gf) by the denier of the yarn. This analysis followed the standard test method ASTM D2256 and was conducted at a room temperature of 20 + 2 °C.

For a robust statistical analysis, these tests were replicated on five specimens at each of the designated time points. The average and standard deviation of the recorded values were calculated to provide a clear indication of the yarn’s mechanical properties during the degradation process.

### 2.4. Statistical Analysis

All data collected during the study are presented in terms of the mean ± standard deviation, derived from five individual data points. To compare the differences in data and ascertain their statistical significance, a student’s t-test was applied using JMP Pro^®^ statistical software (15.2.0, SAS Institute Inc., Cary, NC, USA). Statistical significance was pre-determined at a *p*-value threshold of less than 0.05. Any result yielding a *p*-value less than this threshold was deemed to be statistically significant.

## 3. Results and Discussions

The 5-inch-long PCL multifilament yarn samples were retrieved from the dPBS solution at each time point, namely 8, 16, 24, and 32 weeks. The mass of each 5-inch specimen was measured in milligrams, and the changes in tenacity, breaking force, and elongation at break were monitored over the course of the study. The rationale behind assessing the reduction rates of these properties was to estimate the timeline over which the filaments could maintain their mechanical strength and elongation at break.

**Figure 2:**
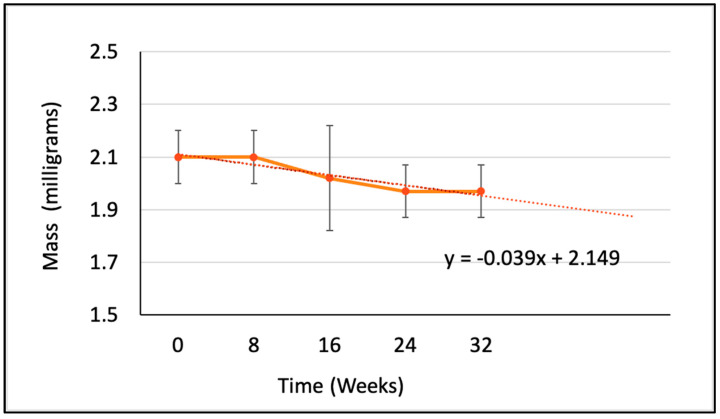
The change in mass in milligrams of the PCL multifilament yarn at each time point over 32 weeks of hydrolytic degradation in dPBS (n = 5).

**Figure 3:**
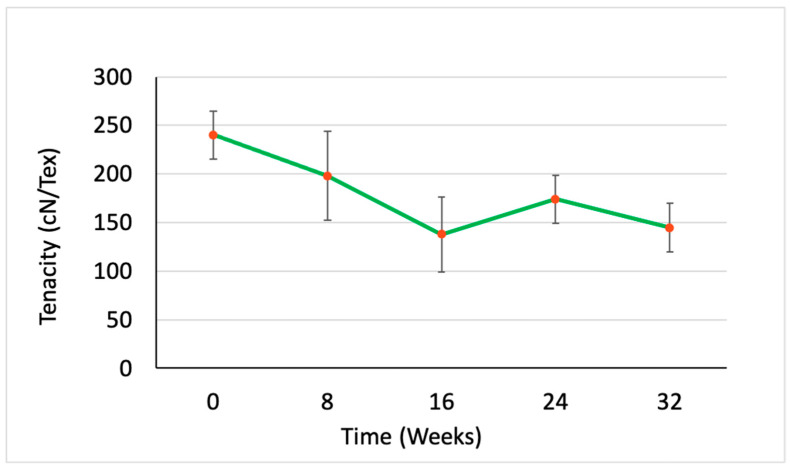
Changes in tenacity in cN per tex of the PCL multifilament yarn at each time point over 32 weeks of hydrolytic degradation in dPBS (n = 5).

**Figure 4:**
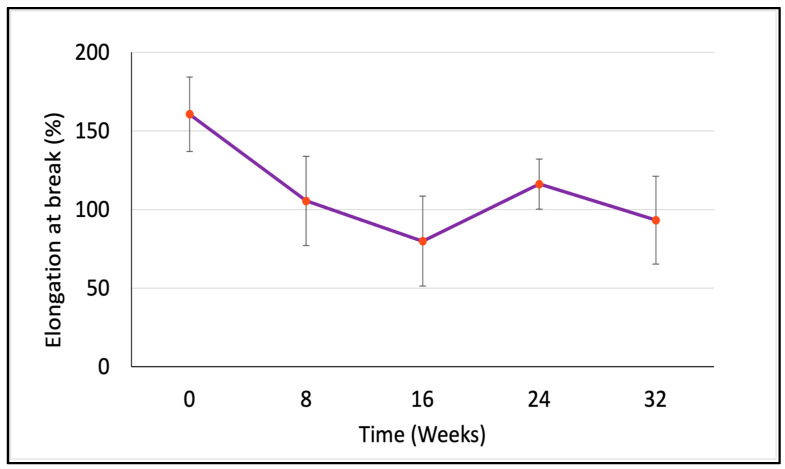
Changes in percent elongation at break of the PCL multifilament yarn at each time point over 32 weeks of hydrolytic degradation in dPBS (n = 5).

**Figure 5:**
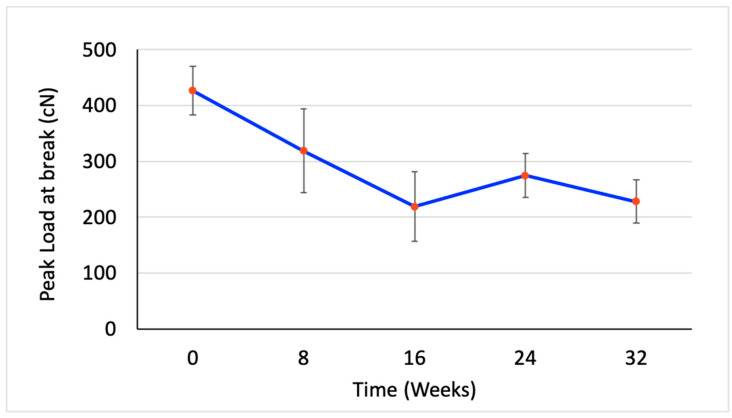
Change in peak load at break in cN of the PCL multifilament yarn at each time point over 32 weeks of hydrolytic degradation in dPBS (n = 5).

The average mass in milligrams, the percentage elongation at break, the peak load in gf, and the tenacity in gf/den were measured and calculated at five time points. However, the measurements were converted into SI units for tenacity (cN/tex) and peak load (cN) as shown in Table 2. Figure 2, Figure 3, Figure 4 and Figure 5 illustrate the changes in these properties plotted against the time of the degradation study in weeks.

Equation (1) was derived from extrapolating the rate of reduction in total mass. This suggests that the PCL filament yarn will take approximately 55.1 weeks to dissolve completely under similar conditions; namely, aqueous solution at pH 7, shaken continuously at 45 rpm, and maintained at 37 ± 2 °C.

(1) y=−0.039x + 2.149

Comparisons were drawn between this study’s results and similar degradation studies on alternative forms of PCL as described in the literature. Over the 32-week period, we observed an approximately 6% loss in mass, a 42% decrease in elongation at break, and about a 40% loss in tenacity. These results are consistent with the findings reported by Lam et al. [8], who studied the changes in mass, maximum stress, crystallinity, and molecular weight during the degradation of PCL in dPBS in a physiological environment.

Figure 2 illustrates the rate of reduction in the total mass of the polymeric filament yarn specimens during the degradation study. During the initial 8 weeks, no significant loss in mass was observed. However, a linear decrease in mass was observed from Week 8 to Week 24, after which the rate of reduction again slowed down. This pattern may reflect the faster degradation of the amorphous regions on the surface of the filaments during the initial stage, followed by a slowed degradation rate as the water molecules took longer to diffuse into the more crystalline structure. This degradation process mirrors that described by Sun et al., who recorded similar stages of PCL capsule degradation in vivo [9].

Figure 3 shows the changes in tenacity of the PCL multifilament yarn during the degradation study. At the start of the study and continuing for the first 16 weeks, the PCL yarn’s tenacity decreased linearly by over 40%. This suggests that the material’s ability to withstand stress was diminishing during this period, likely due to degradation occurring primarily in the less structured, amorphous regions of the polymer. However, between the 16th and 24th weeks, the trend changed direction. Instead of the tenacity continuing to decrease, the yarn experienced an approximate 30% increase in strength. This unexpected enhancement in tenacity could be attributed to the complete degradation of the amorphous regions of the PCL, leaving behind a more crystalline, well-ordered, and aligned structure. A similar trend was observed in the percent elongation at break (Figure 4) and the changes in the peak load at break (Figure 5). The more ordered packing of the polymer chains in the crystalline regions generally imparts a higher tensile strength, which is likely to explain the observed temporary increase in yarn tenacity. After Week 24, the tenacity of the PCL yarn started to decline once more. This final decrease suggests that even the more resistant crystalline regions of the polymer began to degrade under the sustained hydrolytic experimental conditions.

In in vitro and in vivo degradation studies of a 50:50 elastomeric poly(L-lactic acid)/poly(ε-caprolactone) copolymer in the form of an extruded porous tubular scaffold, Jeong et al. observed that the PCL component of the copolymer degraded faster than the PLLA component due to a higher proportion of amorphous regions available for hydrolytic reaction, despite PCL’s greater hydrophobicity [10]. This might explain why the PCL multifilament yarn did not show any structural disintegration throughout the 32-week study and why the total loss in mass was no more than 6%.

PCL, or poly(ε-caprolactone), is a semicrystalline polymer, which means it consists of both amorphous and crystalline regions. The amorphous regions are those where the polymer chains do not have a well-defined, ordered structure, while the crystalline regions are areas where the polymer chains are neatly arranged in a regular, repeating pattern. In this degradation study, PCL multifilament yarn was subjected to hydrolytic conditions for a prolonged period of 32 weeks. As the study progressed, the polymer started to degrade, and the effect of this degradation on the mass, strength, and elasticity of the PCL yarn was measured at different time points. The initial stages of degradation occurred in the amorphous regions of the PCL yarn. Water molecules infiltrate these less-ordered regions more readily, breaking the polymer chains and leading to an initial drop in mass and tensile strength. This explains why the study observed a linear decrease in mass from Week 8 to Week 24.

Once the amorphous regions had substantially degraded, the remaining PCL yarn was more crystalline in nature. At this point, a temporary increase in strength was observed, which was most likely due to the higher tensile strength associated with the ordered crystalline polymer structures. The crystalline regions are more resistant to degradation, hence, the slower rate of mass reduction after 24 weeks. However, eventually, the degradation process also affected the crystalline regions, leading to a further loss in mass and strength over the remaining period of degradation. This slower degradation of the crystalline regions explains why the PCL filament yarn was calculated to take approximately 55.1 weeks to be completely resorbed under the experimental conditions. Overall, both the amorphous and crystalline regions in PCL yarn play a crucial role in determining its degradation profile, which in turn affects its potential application in bioresorbable medical devices.

## 4. Conclusions

This 32-week degradation study provided valuable insights into the behavior of poly(ε-caprolactone) (PCL) multifilament yarn under conditions resembling the physiological environment. Notably, we identified a two-stage degradation process attributable to the semicrystalline nature of PCL. In the initial stage, the amorphous regions of the polymer were rapidly absorbed, followed by a slower degradation of the more ordered crystalline regions.

The study quantitatively documented a linear decrease of over 40% in the tenacity of the yarn up until Week 16, followed by an approximate 30% increase from Weeks 16 to 24, before the tenacity began to decrease again. This fluctuation can be attributed to the increase in the total crystallinity of the yarn as the amorphous regions were fully degraded. The total loss in mass observed during the study was no more than 6%, indicating the material’s resistance to complete degradation during the 32-week experiment.

Extrapolation of the obtained data suggests that full resorption of the PCL multifilament yarn under these conditions will take approximately 14 months. The specific rate and extent of degradation can vary depending on factors such as the exact physiological conditions and the specific design and structure of the implantable device made from the resorbable PCL yarn.

For a more comprehensive understanding of the degradation process, future studies could investigate changes in the average molecular weight and molecular weight distribution of the PCL multifilament yarn during different stages of degradation. This would provide additional evidence to support and possibly refine the degradation profile derived from the present study.

The findings of this study have substantial implications for the design of implantable devices. Understanding the time-dependent mechanical properties and degradation profile of PCL yarn will enable engineers and researchers to adjust their designs so as to predict the in vivo performance and lifespan of implants made from this material. Despite the controlled nature of the experiment, it is acknowledged that actual in vivo conditions may present additional complexities. Therefore, in vitro degradation studies such as this one are pivotal in providing baseline data and should be complemented with in vivo trials for a comprehensive evaluation of the material’s suitability for specific medical applications. The evolving nature of the biomedical industry demands more rigorous, in-depth, and detailed analyses of biodegradable materials. The degradation profile of poly(ε-caprolactone) (PCL) multifilament yarn, as presented in this study, is a pivotal step towards this direction. Future endeavors might shift focus towards understanding the nano-structural transformations and enzymatic interplays that affect the degradation behavior of PCL. This can be further investigated in the blend of materials science and biology to ensure safer, more predictable, and adaptable medical implants for future healthcare needs.

## Figures and Tables

**Table 1:** Physical properties of poly(ε-caprolactone) multifilament yarn as received from the manufacturer.

Polymer	100% Poly(ε-caprolactone)
Approximate average Mw	80 kDa
Denier	160
Number of filaments	36
Elongation at break at room temperature	>150%
Glass transition temperature (Tg)	−60 °C
Melting temperature (Tm)	60 °C
Cross-section	Circular
Storage condition	4 °C

**Table 2:** Average parameters such as mass, percent elongation at break, tenacity, and peak load of the PCL multifilament yarn during 32 weeks of hydrolytic degradation in dPBS.

Yarn Properties	0 Weeks	8 Weeks	16 Weeks	24 Weeks	32 Weeks
Average mass (mg)	2.1 (±0.1)	2.1 (±0.1)	2.0 (±0.2)	2.0 (±0.1)	2.0 (±0.1)
Percent elongation at break (%)	160.7 (±23.7)	105.5 (±28.4)	79.9 (±28.6)	116.2 (±15.8)	93.3 (±28.0)
Tenacity (cN/tex)	240.2 (±24.7)	197.0 (±45.9)	137.7 (±38.8)	174.1 (±24.7)	144.8 (±24.7)
Peak load (cN)	426.4 (±43.7)	319.0 (±74.6)	219.2 (±61.9)	274.6 (±39.3)	228.1 (±38.9)

## Data Availability

All the data supporting the findings of the study are available within the article.
